# An optimized finite element extrapolating method for 2D viscoelastic wave equation

**DOI:** 10.1186/s13660-017-1496-7

**Published:** 2017-09-12

**Authors:** Hong Xia, Zhendong Luo

**Affiliations:** 1 School of Control and Computer Engineering, North China Electric Power University, No. 2, Bei Nong Road, Changping District, Beijing, 102206 China; 2 School of Mathematics and Physics, North China Electric Power University, No. 2, Bei Nong Road, Changping District, Beijing, 102206 China

**Keywords:** 65N15, 65N30, classical finite element method, optimized finite element extrapolating method, proper orthogonal decomposition method, error estimate

## Abstract

In this study, we first present a classical finite element (FE) method for a two-dimensional (2D) viscoelastic wave equation and analyze the existence, stability, and convergence of the FE solutions. Then we establish an optimized FE extrapolating (OFEE) method based on a proper orthogonal decomposition (POD) method for the 2D viscoelastic wave equation and analyze the existence, stability, and convergence of the OFEE solutions and furnish the implement procedure of the OFEE method. Finally, we furnish a numerical example to verify that the numerical computing results correspond with the theoretical ones. This signifies that the OFEE method is feasible and efficient for solving the 2D viscoelastic wave equation.

## Introduction

Let $\Theta\subset\boldsymbol {R}^{2}$ be a bounded convex polygonal domain with a smooth boundary *∂*Θ. We consider the following initial-boundary value problem:

### Problem 1

Seek *u* satisfying

1 $$ \textstyle\begin{cases} u_{tt}-\varepsilon\Delta u_{t}-\gamma\Delta u = f,\quad (x,y,t)\in \Theta\times(0,T], \\u(x,y,t)=\varphi(x,y,t),\quad(x,y,t)\in\partial\Theta\times(0, T], \\u(x,y,0)=\varphi_{0}(x, y),\qquad u_{t}(x,y,0)=\varphi_{1}(x,y),\quad (x, y)\in \Theta, \end{cases} $$

where $u_{tt}={\partial^{2} u}/{\partial t^{2}}$, $u_{t}={\partial u}/{\partial t,}$ and *ε* and *γ* are two positive constants, $f(x,y,t)$, $\varphi(x,y,t)$, and $\varphi_{0}(x, y)$ and $\varphi_{1}(x, y)$ are, respectively, the source term, the boundary value function, and the initial value functions, sufficiently smooth to ensure the validity of the following analysis, and *T* is the time duration. As a matter of convenience, we assume that $\varphi (x,y,t)=0$ and $\varepsilon=\gamma=1$ in the remaining part of the article.

Problem 1 is referred to as a system of viscoelastic wave equations. It has some special and significant physical backgrounds. For instance, it can be used to describe the wave propagation phenomena of actual vibration through a viscoelastic medium (see, *e.g.*, [1, 2]). Although the existence and uniqueness of its analytic solution have been proved (see, *e.g.*, [3–6]), because the viscoelastic wave equation in the real-world engineering applications usually has complex known data or computed domains, the analytical solution cannot be generally solved, so one has to find its solutions numerically. For more than 30 years, it has been attentively studied and many numerical methods for the viscoelastic wave equation have been developed (see, *e.g.*, [5–8]). Among all numerical methods, the finite element (FE) method is considered to be one of the calculating numerical methods with the best theory for the two-dimensional (2D) viscoelastic wave equation (see [8, 9]). Nevertheless, the classical FE methods for the 2D viscoelastic wave equation are some macroscale systems of equations including lots of unknowns, *i.e.*, degrees of freedom, so entail very large computational load in real-world engineering applications. As a consequence, an important issue is how to greatly lessen the number of unknowns of the classical FE methods to reduce the computational load, ease the truncated error amassing, and save CPU time in the numerical computation, while preserving the desired FE solution accuracy.

It has been proved by lots of numerical studies (see, *e.g.*, [10–23]) that the proper orthogonal decomposition (POD) method is a very useful tool to reduce the number of unknowns for numerical models and ease the truncated error amassing in numerical calculations. But most existing reduced-order models, as mentioned, were established via the POD basis formed from the classical numerical solutions at all time nodes, before repetitively computing the reduced-order numerical solutions at the same time nodes, which were some valueless repetitive calculations. Since 2014, some reduced-order FE extrapolating methods based on the POD method for partial differential equations have been established successively by Luo’s team (see, *e.g.*, [24–26]) in order to avert the valueless repeated computations.

However, as far as we know, there has not been any report that the POD method is used to reduce the number of unknowns in the classical FE method for the 2D viscoelastic wave equation. Therefore, in this article, we devote ourselves to building an optimized FE extrapolating (OFEE) method that includes very few unknowns but maintains desired accuracy via the POD method, analyzing the existence, stability, and convergence of the OFEE solutions and verifying the efficiency and feasibility of the OFEE method by some numerical simulations.

The main distinctions between the OFEE method and the other existing reduced-order FE extrapolating methods built on the POD method (see, *e.g.*, [24–26]) consist in the fact that the viscoelastic wave equation not only contains three second-order derivative terms of time and of spatial variables but also includes two mixed derivative terms of time (first-order) and spatial variables (second-order) so that either the modeling of the OFEE method or the demonstration of the existence, stability, and convergence of the OFEE solutions faces more difficulties and requires more techniques than the existing other aforementioned reduced-order FE extrapolating methods. However, the OFEE method has some specific applications. Though an optimized splitting positive definite mixed FE extrapolation (OSPDMFEE) model based on the POD technique for the 2D viscoelastic wave equation is developed in [27], it has three unknown functions and the OSPDMFEE model has more degrees of freedom than the current OFEE format, so that its theoretical analysis and numerical simulations have more difficulties than the current OFEE method. It is worth mentioning that we can discuss the existence, stability, and convergence of the reduced-order FE solutions by means of the classical FE theory. Especially, the OFEE method only employs the classical FE solutions at the initial very few time nodes to formulate the POD basis and build the OFEE format so that it does not have repetitive calculations, such as done in references [24–27]. Consequently, it is a development and an improvement of the existing aforementioned ones (see, *e.g.*, [10–23]).

The remaining content of the article is organized as follows. In Section 2, we first present the classical FE method for the 2D viscoelastic wave equation and analyze the existence, stability, and convergence of the classical FE solutions. In Section 3, we develop the OFEE method via the POD method for the 2D viscoelastic wave equation, analyze the stability and convergence of the OFEE solutions, and furnish the implement procedure of the OFEE method. Next, in Section 4, we use some numerical simulations to verify the efficiency and feasibility of the OFEE method. Finally, in Section 5, we summarize our main conclusions.

## The classical FE method for the 2D viscoelastic wave equation

### Generalized solution for the 2D viscoelastic wave equation

The following arisen Sobolev spaces as well as their norms are well known (see [28]).

For convenience, we write $U=H^{1}_{0}(\Theta)$. Thus, by using Green’s formula for the 2D viscoelastic wave equation, we obtain the following variational formulation:

#### Problem 2

For $t\in(0, T)$, seek $u\in U$ satisfying

2 $$\begin{aligned}& (u_{tt},v)+(\nabla u_{t},\nabla v)+( \nabla u,\nabla v) = (f,v),\quad\forall v\in U, \end{aligned}$$

3 $$\begin{aligned}& u(x,y,0)=\varphi_{0}(x, y),\qquad u_{t}(x,y,0)= \varphi_{1}(x,y),\quad(x, y)\in\Theta, \end{aligned}$$

where $(\cdot,\cdot)$ denotes the inner product of $L^{2}(\Theta)$.

For *U*, we have the following Poincaré inequality:

$$\|\nabla u\|_{0}\leq\|u\|_{1}\leq\beta\|\nabla u \|_{0}, \quad\forall u\in U, $$

where *β* is a positive real.

For Problem 2, we have the following result.

#### Theorem 1

*If* $f\in H^{-1}(\Theta)$, $\varphi_{1}(x, y)\in L^{2}(\Theta)$, *and* $\varphi_{0}(x,y)\in H^{1}(\Theta)$, *then Problem* 2 *has a unique solution* $u\in H^{1}_{0}(\Theta)$ *satisfying*

4 $$ \|u_{t} \|_{0}^{2}+ \int_{0}^{t}\|\nabla u_{t} \|_{0}^{2}\,\mathrm{d}t+\|\nabla u\|_{0}^{2} \leq \beta^{-2} \int_{0}^{t}\|f\|_{-1}^{2}\,\mathrm{d}t+\| \varphi_{1}\|_{0}^{2}+\big\| \nabla\varphi _{0}(x, y)\big\| _{0}^{2}, $$

*where* *β* *is the constant in the Poincaré inequality*.

#### Proof

Because Problem 2 is a system of linear equations as regards the unknown function *u*, in order to prove the existence and uniqueness of solutions for Problem 2, it is necessary to prove that Problem 2 has only the zero solution when $f(x,y,t)=\varphi _{0}(x, y)=\varphi_{1}(x,y)=0$.

By taking $v=u_{t}$ in (2), we have

5 $$ (u_{tt},u_{t})+(\nabla u_{t}, \nabla u_{t})+(\nabla u,\nabla u_{t}) = (f,u_{t}). $$

Thus, by the Hölder inequality, the Poincaré inequality, and the Cauchy-Schwarz inequality, we acquire

6 $$ \frac{\mathrm{d}\|u_{t}\|_{0}^{2}}{2\,\mathrm{d}t}+\|\nabla u_{t} \|_{0}^{2}+\frac {\mathrm{d}\|\nabla u\|_{0}^{2}}{2\,\mathrm{d}t}\leq\beta^{-1}\|f \|_{-1}\|\nabla u_{t}\|_{0} \leq\frac{\|f\|_{-1}^{2}}{2\beta^{2}}+ \frac{\|\nabla u_{t}\|_{0}^{2}}{2}. $$

By integrating (6) from 0 to $t\in[0,T]$, we obtain

7 $$ \|u_{t}\|_{0}^{2}+ \int_{0}^{t}\|\nabla u_{t} \|_{0}^{2}\,\mathrm{d}t+\|\nabla u\|_{0}^{2} \leq \beta^{-2} \int_{0}^{t}\|f\|_{-1}^{2}\,\mathrm{d}t+\| \varphi_{1}\|_{0}^{2}+\big\| \nabla\varphi_{0}(x, y)\big\| _{0}^{2}, $$

which is the stated inequality (4). Thus, when $f(x,y,t)=\varphi_{0}(x, y)=\varphi_{1}(x,y)=0$, from (7), we obtain $\|u_{t}\|_{0}=\|\nabla u_{t}\|_{0}=\|\nabla u\|_{0}=0$, which implies $u=0$. Then Problem 2 has a unique solution such that inequality (4) holds. □

### Semi-discrete format as regards time for the 2D viscoelastic wave equation

Let *N* be a positive integer, $\Delta t=T/N$ the time step size, and $t_{i}=i\Delta t$. If we use $(u^{n+1}-u^{n})/ (2\Delta t)$ to approximate $u_{t}$ and $(u^{n+1}-2u^{n}+u^{n-1})/\Delta t^{2}$ to approximate $u_{tt}$ for the 2D viscoelastic wave equation, we obtain the following semi-discrete formulation of time:

#### Problem 3

Seek $u^{n+1}\in U$ satisfying

8 $$\begin{aligned}& \begin{aligned}[b]&\frac{1}{\Delta t^{2}} \bigl(u^{n+1}-2u^{n}+u^{n-1},v\bigr)+\frac{1}{2\Delta t} \bigl(\nabla\bigl(u^{n+1}-u^{n-1}\bigr),\nabla v\bigr) \\ &\quad{}+\frac{1}{2}\bigl(\nabla\bigl(u^{n+1}+u^{n-1} \bigr),\nabla v\bigr) = \bigl(f^{n},v\bigr),\quad\forall v\in U, n=1,2, \ldots,N-1, \end{aligned} \end{aligned}$$

9 $$\begin{aligned}& u^{0}=\varphi_{0}(x, y),\qquad u^{1}=\varphi_{0}(x, y)+\Delta t\varphi_{1}(x,y),\quad (x, y)\in\Theta, \end{aligned}$$

where $f^{n}=f(t_{n})$.

For Problem 3, we have the following.

#### Theorem 2

*Under the assumptions of Theorem* 1, *if* $\varphi_{0}, \varphi_{1} \in H^{1}_{0}(\Theta)$, *then Problem* 3 *has a unique solution* $u^{n}\in U$ *satisfying*

10 $$ \big\| \nabla u^{n}\big\| _{0} \leq\Biggl({\beta^{-2}} {\Delta t}\sum_{i=1}^{n} \| f^{i}\|_{-1}^{2}+ \|\nabla\varphi_{0} \|_{0}^{2}+\|\varphi_{1}\|_{1}^{2} \Biggr)^{1/2},\quad n=1, 2, \ldots, N, $$

*showing that the series of solutions to Problem* 3 *is stable and continuously dependent on the source function* *f* *and the initial values* $\varphi_{0}$ *and* $\varphi_{1}$. *When* *u* *is sufficiently smooth in* *t*, *we have the following error estimations*:

11 $$ \big\| \nabla\bigl(u^{n}-u(t_{n}) \bigr)\big\| _{0}\leq C_{0}\Delta t^{2},\quad n=1,2,\ldots, N, $$

*where* $C_{0}^{2}=\frac{T}{48\beta^{2}}\|u^{(4)}(\xi_{1}^{n})\|_{-1}^{2} +\frac {T}{12}\|\nabla u_{ttt}(\xi_{2}^{n})\|_{0}^{2}+\frac{3T}{16}\|\nabla u_{tt}(\xi_{3}^{n})\|_{0}^{2}$ ($t_{n-1}\leq\xi_{1}^{n}, \xi_{2}^{n}, \xi _{3}^{n}\leq t_{n+1}$).

#### Proof

Because Problem 3 is a system of linear equations as regards the unknown function $u^{n}$, in order to prove the existence and uniqueness of solutions for Problem 3, it is necessary to prove that Problem 3 has only the set of zero solutions when $f(x,y,t)=\varphi_{0}(x, y)=\varphi_{1}(x,y)=0$.

By taking $v=u^{n+1}-u^{n-1}$ in (8) and using the Hölder, Poincaré, and Cauchy-Schwarz inequalities, we have

12 $$ \begin{aligned}[b] &\big\| u^{n+1}-u^{n} \big\| _{0}^{2}-\big\| u^{n}-u^{n-1} \big\| _{0}^{2}+\frac{\Delta t}{2}\big\| \nabla\bigl(u^{n+1}-u^{n-1} \bigr)\big\| _{0}^{2} \\ &\qquad{}+\frac{\Delta t^{2}}{2}\bigl(\big\| \nabla u^{n+1} \big\| _{0}^{2}-\big\| \nabla u^{n-1}\big\| _{0}^{2} \bigr) \\ &\quad\leq\frac{\Delta t^{3}}{2\beta^{2}}\big\| f^{n} \big\| _{-1}^{2}+\frac{\Delta t}{2}\big\| \nabla\bigl(u^{n+1}-u^{n-1} \bigr)\big\| _{0}^{2}. \end{aligned} $$

By summing (12) from 1 to *n* and using (9), we obtain

13 $$ \begin{aligned}[b] &\big\| u^{n+1}-u^{n} \big\| _{0}^{2}+{\Delta t^{2}}\bigl(\big\| \nabla u^{n+1}\big\| _{0}^{2}+\big\| \nabla u^{n} \big\| _{0}^{2}\bigr) \\ &\quad\leq\frac{\Delta t^{3}}{\beta^{2}}\sum_{i=1}^{n} \big\| f^{i}\big\| _{-1}^{2}+\Delta t^{2}\bigl(\| \nabla\varphi_{0}\|_{0}^{2}+\|\nabla \varphi_{1}\|_{0}^{2}\bigr)+2\Delta t^{2} \| \varphi_{1}\|_{0}^{2}. \end{aligned} $$

Thus, when $f(x,y,t)=\varphi_{0}(x, y)=\varphi_{1}(x,y)=0$, from (13), we obtain $\|\nabla u^{n}\|_{0}=0$, implying $u^{n}=0$. Hence Problem 3 has a unique solution series.

From (13), we obtain

14 $$ \big\| \nabla u^{n} \big\| _{0}^{2}\leq{\beta^{-2}}\Delta t\sum _{i=1}^{n}\big\| f^{i}\big\| _{-1}^{2}+ \|\nabla\varphi_{0}\|_{0}^{2}+\| \varphi_{1}\|_{1}^{2}. $$

From (14), we obtain

15 $$ \big\| \nabla u^{n}\big\| _{0} \leq\Biggl({\beta^{-2}} {\Delta t}\sum_{i=1}^{N} \big\| f^{i}\big\| _{-1}^{2}+ \|\nabla\varphi_{0} \|_{0}^{2}+\|\varphi_{1}\|_{1}^{2} \Biggr)^{1/2}, $$

which is just the inequality (10).

Let $e^{n}=u(t_{n})-u^{n}$. By applying the Taylor expansion formula to (8) and then subtracting (2) taking $t=t_{n}$, we obtain

16 $$ \begin{aligned}[b] &\bigl(e^{n+1}-2e^{n}-e^{n-1},v \bigr)+\frac{\Delta t}{2}\bigl(\nabla\bigl(e^{n+1}-e^{n-1} \bigr),\nabla v\bigr)+\frac{1}{2}\bigl(\nabla e^{n+1}+\nabla e^{n-1}, \nabla v\bigr) \\ &\quad=\frac {\Delta t^{4}}{12}\bigl(u^{(4)}\bigl( \xi_{1}^{n}\bigr),v\bigr) +\frac{\Delta t^{4}}{6}\bigl(\nabla u_{ttt}\bigl( \xi_{2}^{n}\bigr),\nabla v\bigr)+\frac {\Delta t^{4}}{4}\bigl( \nabla u_{tt}\bigl(\xi_{3}^{n}\bigr),\nabla v\bigr), \end{aligned} $$

where $t_{n-1}\leq\xi_{1}^{n}, \xi_{2}^{n}, \xi_{3}^{n}\leq t_{n+1}$. By taking $v=e^{n+1}-e^{n-1}$ in (16), we obtain

17 $$ \begin{aligned}[b] &\big\| e^{n+1}-e^{n} \big\| _{0}^{2}-\big\| e^{n}-e^{n-1} \big\| _{0}^{2}+\frac{\Delta t}{2}\big\| \nabla\bigl(e^{n+1}-e^{n-1} \bigr)\big\| _{0}^{2}+\frac{\Delta t^{2}}{2}\bigl(\big\| \nabla e^{n+1} \big\| _{0}^{2}-\big\| \nabla e^{n-1}\big\| _{0}^{2} \bigr) \\ &\quad=\frac{\Delta t^{4}}{4}\bigl(\nabla u_{tt}(\xi_{3}),\nabla \bigl(e^{n+1}-e^{n-1}\bigr)\bigr)+\frac{\Delta t^{4}}{6}\bigl(\nabla u_{ttt}( \xi_{2}),\nabla\bigl(e^{n+1}-e^{n-1}\bigr)\bigr) \\ &\qquad{}+ \frac{\Delta t^{4}}{12}\bigl(u^{(4)}(\xi_{1}),e^{n+1}-e^{n-1} \bigr) \\ &\quad\leq\frac{\Delta t}{2}\big\| \nabla\bigl(e^{n+1}-e^{n-1} \bigr)\big\| _{0}^{2}+\frac{\Delta t^{7}}{96\beta^{2}}\big\| u^{(4)}\bigl( \xi_{1}^{n}\bigr)\big\| _{-1}^{2} + \frac{\Delta t^{7}}{24}\big\| \nabla u_{ttt}\bigl(\xi_{2}^{n} \bigr)\big\| _{0}^{2} \\ &\qquad{}+\frac{3\Delta t^{7}}{32}\big\| \nabla u_{tt}\bigl( \xi_{3}^{n}\bigr)\big\| _{0}^{2}, \end{aligned} $$

where *β* is the same constant as in the Poincaré inequality. Because $e^{0}=0$, $e^{1}=0$ (when Δ*t* is sufficiently small), by summing (17) from 1 to *n*, we obtain

18 $$ \big\| e^{n+1}-e^{n} \big\| _{0}^{2}+{\Delta t^{2}}\bigl(\big\| \nabla e^{n+1}\big\| _{0}^{2}+\big\| \nabla e^{n} \big\| _{0}^{2}\bigr) \leq\Delta t^{6}C_{0}^{2}, $$

where $C_{0}^{2}=\frac{T}{48\beta^{2}}\|u^{(4)}(\xi_{1}^{n})\|_{-1}^{2} +\frac {T}{12}\|\nabla u_{ttt}(\xi_{2}^{n})\|_{0}^{2}+\frac{3T}{16}\|\nabla u_{tt}(\xi_{3}^{n})\|_{0}^{2}$. From (18), we obtain

19 $$ \big\| \nabla e^{n}\big\| _{0} \leq\Delta t^{2}C_{0}. $$

This finishes the proof of Theorem 2. □

### Classical fully discrete FE method for the 2D viscoelastic wave equation

Let $\Im_{h}$ be a regular triangulation of Θ̄. The FE subspace $U_{h}$ is taken as

20 $$ U_{h}= \bigl\{ v_{h}\in U\cap C(\bar{ \Theta}): v_{h}|_{K}\in\mathcal{P}_{k}(K), \forall K\in\Im_{h} \bigr\} , $$

where $\mathcal{P}_{k}(K)$ is the subspace formed by *k*th degree polynomials on *K* and $k\geq1$ is an integer.

Thus, the fully discrete FE formulation for the 2D viscoelastic wave equation (1) is as follows:

#### Problem 4

Seek $u_{h}^{n+1}\in U_{h}$ ($n=1,2,\ldots,N-1$) satisfying

21 $$\begin{aligned}& \begin{aligned}[b] & \frac{1}{\Delta t^{2}} \bigl(u_{h}^{n+1}-2u_{h}^{n}+u_{h}^{n-1},v_{h} \bigr)+\frac {1}{2\Delta t}\bigl(\nabla\bigl(u_{h}^{n+1}-u_{h}^{n-1} \bigr),\nabla v_{h}\bigr) \\ &\quad{} +\frac{1}{2}\bigl(\nabla\bigl(u_{h}^{n+1}+u_{h}^{n-1} \bigr),\nabla v_{h}\bigr) = \bigl(f^{n},v_{h} \bigr),\quad\forall v_{h}\in U_{h}, 1\leq n\leq N-1, \end{aligned} \end{aligned}$$

22 $$\begin{aligned}& u_{h}^{0}=R_{h} \varphi_{0}(x, y),\qquad u_{h}^{1}=R_{h}\bigl( \varphi_{0}(x, y)\bigr)+\Delta tR_{h}\bigl( \varphi_{1}(x,y)\bigr), \quad(x, y)\in\Theta, \end{aligned}$$

where $f^{n}=f(t_{n})$ and $R_{h}$ is the Ritz projection as follows:

$$\bigl(\nabla(\varphi_{i}-R_{h}\varphi_{i}), \nabla v_{h}\bigr)=0, \quad\forall v_{h}\in U_{h}, i=0,1. $$

For Problem 4, we have the following.

#### Theorem 3

*Under the assumptions of Theorems* 2 *and* 3, *Problem* 4 *has a unique solution set* $\{u_{h}^{n}\}_{n=1}\subset U_{h}$ *satisfying*

23 $$ \big\| \nabla u_{h}^{n} \big\| _{0} \leq\Biggl({\beta^{-2}}\Delta t\sum_{i=1}^{n}\big\| f^{i}\big\| _{-1}^{2}+ 2\|\nabla \varphi_{0}\|_{0}^{2}+ \bigl(2+\beta^{-2}\bigr)\|\nabla\varphi_{1} \|_{1}^{2} \Biggr)^{1/2}. $$

*Consequently*, *the solution sequence* $u_{h}^{n}$ *to Problem* 4 *is stable and continuously dependent on the source function* *f* *and the initial values* $\varphi_{0}$ *and* $\varphi_{1}$. *With* $h=O(\Delta t)$, *we have the following error estimations*:

24 $$ \big\| \nabla\bigl(u_{h}^{n}-u(t_{n}) \bigr)\big\| _{0}\leq C\bigl(\Delta t^{2}+h^{k}\bigr),\quad n=1,2,\ldots, N, $$

*where* *C* *is a positive constant only dependent on* *u*, *but independent of the time step* Δ*t* *and spatial mesh parameters* *h*.

#### Proof

(i) *The existence and uniqueness of the solution sequence for Problem* *4*.

Let

$$a\bigl(u_{h}^{n+1},v_{h}\bigr)= 2 \bigl(u_{h}^{n+1},v_{h}\bigr)+\Delta t\bigl(\nabla u_{h}^{n+1},\nabla v_{h}\bigr)+ \Delta t^{2}\bigl(\nabla u_{h}^{n+1},\nabla v_{h}\bigr) $$

and

$$F(v_{h})={\Delta t^{2}}\bigl(f^{n},v_{h} \bigr)+2\bigl(2u_{h}^{n}-u_{h}^{n-1},v_{h} \bigr)+{\Delta t}\bigl(\nabla u_{h}^{n-1},\nabla v_{h}\bigr)-{\Delta t}^{2}\bigl(\nabla u_{h}^{n-1}, \nabla v_{h}\bigr). $$

Then Problem 4 can be rewritten as follows:

#### Problem 5

Seek $u_{h}^{n+1}\in U_{h}$ ($n=1,2,\ldots,N-1$) satisfying

25 $$\begin{aligned}& a\bigl(u^{n+1}_{h},v_{h} \bigr)=F(v_{h}),\quad\forall v_{h}\in U_{h}, 1\leq n\leq N-1, \end{aligned}$$

26 $$\begin{aligned}& u_{h}^{0}=R_{h}\varphi_{0}(x, y),\qquad u_{h}^{1}=R_{h}\bigl(\varphi_{0}(x, y)\bigr)+\Delta tR_{h}\bigl(\varphi_{1}(x,y)\bigr),\quad(x, y) \in\Theta. \end{aligned}$$

It is obvious that, for given $u_{h}^{n}$ and $u_{h}^{n-1}$ as well as $f^{n}$ ($n=1,2, \ldots, N-1$), $F(v_{h})$ is a bounded linear functional of $v_{h}$ and $a(u,v)$ is a bilinear functional of *u* and *v*. Because $\|u\|_{0}\leq\|u\|_{1}$ and $\|\nabla u\|_{0}\leq\|u\|_{1}$, by using the Hölder inequality, we have

$$\begin{aligned}\big| a\bigl(u_{h}^{n+1},v_{h} \bigr)\big|&= \big|2\bigl(u_{h}^{n+1},v_{h}\bigr)+ \Delta t\bigl(\nabla u_{h}^{n+1},\nabla v_{h} \bigr)+ \Delta t^{2}\bigl(\nabla u_{h}^{n+1},\nabla v_{h}\bigr)\big| \\ &\leq2\big\| u_{h}^{n+1}\big\| _{0} \|v_{h}\|_{0}+\Delta t\big\| \nabla u_{h}^{n+1} \big\| _{0}\|\nabla v_{h}\|_{0}+ \Delta t^{2} \big\| \nabla u_{h}^{n+1}\big\| _{0}\|\nabla v_{h} \|_{0} \\ &\leq M\big\| u_{h}^{n+1}\big\| _{1} \|v_{h}\|_{1}, \end{aligned} $$

where $M=\max\{2, \Delta t, \Delta t^{2}\}$. Therefore, $a(u,v)$ is bounded in $U_{h}\times U_{h}$. Furthermore, we have

27 $$ \begin{aligned}[b] a(v,v)&= 2(v,v)+\Delta t( \nabla v,\nabla v)+ \Delta t^{2}(\nabla v,\nabla v) \\ &=2\|v\|_{0}^{2}+\Delta t\|\nabla v \|_{0}^{2}+ \Delta t^{2}\|\nabla v \|_{0}^{2} \\ &\geq\alpha\|v\|_{1}^{2},\quad\forall v\in U_{h}, \end{aligned} $$

where $\alpha=\min\{2, \Delta t, \Delta t^{2}\}$. Thus, it is positive definitive on $U_{h}\times U_{h}$. Therefore, by the Lax-Milgram theorem, Problem 5 and also Problem 4 have a unique solution sequence $\{u_{h}^{n}\}_{n=1}^{N}$.

(ii) *The stability of the solution sequence* $\{u_{h}^{n}\}_{n=1}^{N}$ *for Problem* *4* *, i.e., inequality* (*23*).

By taking $v_{h}=u_{h}^{n+1}-u_{h}^{n-1}$ in (21) and using the Hölder, Poincaré, and Cauchy-Schwarz inequalities, we have

28 $$ \begin{aligned}[b] &\big\| u_{h}^{n+1}-u_{h}^{n} \big\| _{0}^{2}-\big\| u_{h}^{n}-u_{h}^{n-1} \big\| _{0}^{2}+\frac{\Delta t}{2}\big\| \nabla\bigl(u_{h}^{n+1}-u_{h}^{n-1} \bigr)\big\| _{0}^{2} \\ &\qquad{}+\frac{\Delta t^{2}}{2}\bigl(\big\| \nabla u_{h}^{n+1} \big\| _{0}^{2}-\big\| \nabla u_{h}^{n-1} \big\| _{0}^{2}\bigr) \\ &\quad\leq\frac{\Delta t^{3}}{2\beta^{2}}\big\| f^{n} \big\| _{-1}^{2}+\frac{\Delta t}{2}\big\| \nabla\bigl(u_{h}^{n+1}-u_{h}^{n-1} \bigr)\big\| _{0}^{2}. \end{aligned} $$

By summing (28) from 1 to *n* and using (22), again the Poincaré inequality, and the properties of the Ritz projection $R_{h}$, we obtain

29 $$ \begin{aligned}[b] &\big\| u_{h}^{n+1}-u_{h}^{n} \big\| _{0}^{2}+{\Delta t^{2}}\bigl(\big\| \nabla u_{h}^{n+1}\big\| _{0}^{2}+\big\| \nabla u_{h}^{n}\big\| _{0}^{2}\bigr) \\ &\quad\leq \frac{\Delta t^{3}}{\beta^{2}}\sum_{i=1}^{n}\big\| f^{i}\big\| _{-1}^{2}+2\Delta t^{2}\bigl(\|\nabla \varphi_{0}\|_{0}^{2}+\|\nabla\varphi_{1} \| _{0}^{2}\bigr)+\beta^{-2}\Delta t^{2} \|\nabla\varphi_{1}\|_{0}^{2}. \end{aligned} $$

From (29), we immediately obtain (23).

(iii) *Convergence of the solution sequence for Problem* *4*.

Let $\tilde{e}^{n}=u^{n}-u_{h}^{n}$, $E^{n}=R_{h}u^{n}-u_{h}^{n}$, and $\rho ^{n}=u^{n}-R_{h}u^{n}$. By subtracting Problem 4 from Problem 3, taking $v=v_{h}\in U_{h}$, we obtain the following system of the error equations:

30 $$\begin{aligned}& \begin{aligned}[b] & \frac{1}{\Delta t^{2}}\bigl( \tilde{e}^{n+1}-2\tilde{e}^{n}+\tilde{e}^{n-1},v_{h} \bigr) +\frac{1}{2\Delta t}\bigl(\nabla\bigl(\tilde{e}^{n+1}-\tilde{e}^{n-1}\bigr),\nabla v_{h}\bigr) \\ &\qquad{}+\frac{1}{2}\bigl(\nabla\bigl(\tilde{e}^{n+1}+ \tilde{e}^{n-1}\bigr),\nabla v_{h}\bigr) = 0,\quad \forall v_{h}\in U_{h}, 1\leq n\leq N-1, \end{aligned} \end{aligned}$$

31 $$\begin{aligned}& \tilde{e}^{0}=\rho^{0},\qquad \tilde{e}^{1}=\rho^{0}+\Delta t\bigl[\varphi _{1}(x,y)-R_{h}\bigl(\varphi_{1}(x,y)\bigr)\bigr],\quad (x, y)\in\Theta. \end{aligned}$$

By (30) and the properties of the Ritz projection $R_{h}$, when $h=O(\Delta t)$, we have

32 $$ \begin{aligned}[b] &\big\| E^{n+1}-E^{n} \big\| _{0}^{2}-\big\| E^{n}-E^{n-1} \big\| _{0}^{2}+\frac{\Delta t}{2}\big\| \nabla\bigl(E^{n+1}-E^{n-1} \bigr)\big\| _{0}^{2} \\ &\qquad{}+\frac{\Delta t^{2}}{2}\bigl(\big\| \nabla E^{n+1} \big\| _{0}^{2}-\big\| \nabla E^{n-1}\big\| _{0}^{2} \bigr) \\ &\quad=-\bigl(\rho^{n+1}-2\rho^{n}+ \rho^{n-1},E^{n+1}-E^{n-1}\bigr) \\ &\quad\leq Ch^{-1}\bigl(\big\| \rho^{n+1} \big\| _{-1}^{2}+\big\| \rho^{n}\big\| _{-1}^{2}+ \big\| \rho^{n-1}\big\| _{-1}^{2}\bigr) +\frac{\Delta t}{2}\big\| \nabla\bigl(E^{n+1}-E^{n-1}\bigr)\big\| _{0}^{2} \\ &\quad\leq Ch^{2k+3}+\frac{\Delta t}{2}\big\| \nabla \bigl(E^{n+1}-E^{n-1}\bigr)\big\| _{0}^{2}. \end{aligned} $$

By summing (32) from 1 to *n*, we obtain

33 $$ \begin{aligned}[b] &\big\| E^{n+1}-E^{n} \big\| _{0}^{2}+\frac{\Delta t^{2}}{2}\bigl(\big\| \nabla E^{n+1} \big\| _{0}^{2}+\big\| \nabla E^{n}\big\| _{0}^{2} \bigr) \\ &\quad\leq CTh^{2k+2}+\big\| E^{1}-E^{0} \big\| _{0}^{2}+\frac{\Delta t^{2}}{2}\bigl(\big\| \nabla E^{1} \big\| _{0}^{2}+\big\| \nabla E^{0}\big\| _{0}^{2} \bigr) \\ &\quad\leq Ch^{2k}(\Delta t)^{2}. \end{aligned} $$

From (33), by the properties of the Ritz projection and Theorem 2, we immediately obtain (24). □

#### Remark 1

The full FE formulation Problem 4 is directly built from the semi-discrete formulation Problem 3 with respect to time such that one can bypass the semi-discrete formulation with respect to spatial variables and its theoretical analysis becomes simpler. Thus, as long as $f(x,y,t)$, $\varphi_{0}(x, y)$, $\varphi_{1}(x, y)$, *ε*, *γ*, time step *k*, the spatial mesh size *h*, and the FE subspace $U_{h}$ are assigned, we attain the solution sequence $\{u_{h}^{n} \}_{n=1}^{N}\subset U_{h}$ by solving Problem 4. We take the subsequence $\{u_{h}^{n}\} _{n=1}^{L}$ from the initial *L* solutions of $\{u_{h}^{n} \}_{n=1}^{N}$ as snapshots (in general, $L\ll N$ and $\sqrt{L}<5$, for example, $L=20$, $N=200$).

## The OFEE format for the 2D viscoelastic wave equation

### Formulations of the POD basis and establishment the OFEE format

Let $W_{n}(x,y)= u_{h}^{n}(x,y)$ ($1\leq n\leq L$), at least one of which is supposed to be a non-zero function, and $l=\operatorname{dim}\{W_{1}, W_{2},\ldots,W_{L} \}$. Write ${\boldsymbol {A}}=({A}_{ik})_{L\times L}$ and ${A}_{ik}=(\nabla{W}_{i}(x,y),\nabla{W}_{k}(x,y))/L$. Since the matrix ***A*** is a non-negative Hermitian matrix with rank *l*, it has a complete set of orthonormal eigenvectors

34 $$ \begin{gathered}{\boldsymbol {v}}^{1}= \bigl( a_{1}^{1}, a_{2}^{1}, \ldots, a_{L} ^{1} \bigr)^{T},\qquad{\boldsymbol {v}}^{2}= \bigl(a_{1}^{2}, a_{2}^{2}, \ldots, a_{L} ^{2} \bigr)^{T},\qquad\ldots,\\ {\boldsymbol {v}}^{L} = \bigl(a_{1}^{L} , a_{2}^{L} , \ldots, a_{L} ^{L} \bigr)^{T} \end{gathered} $$

with corresponding eigenvalues $\lambda_{1}\geq\lambda_{2}\geq\cdots \geq\lambda_{L} >0$. Thus, the POD basis $\{{\psi}_{1},{\psi}_{2},\ldots, {\psi}_{L} \} $ is given by (see [17])

35 $$ {\psi}_{j}=\frac{1}{\sqrt{ L \lambda_{j}}}\sum _{i=1}^{L} a_{i}^{j}{W}_{i},\quad 1\leq j\leq d\leq l, $$

holding the following property (see also [17]).

#### Proposition 4

*The following estimation holds*:

36 $$ \frac{1}{ L }\sum_{i=1}^{L} \Bigg\| {W}_{i}- \sum_{j=1}^{d}({W}_{i},{ \psi}_{j})_{U}{ \psi}_{j}\Bigg\| ^{2}_{U}= \sum_{j=d+1}^{l}\lambda_{j}. $$

Let $U^{d}=\hbox{span} \{\psi_{1},\psi_{2}, \ldots, \psi_{d} \}$. For $u_{h}\in U_{h}$, formulate the Ritz-operator $R^{d}: U_{h}\to U^{d}$ by

37 $$ \bigl(\nabla R^{d}u_{h}, \nabla w_{d}\bigr)= (\nabla u_{h}, \nabla w_{d}),\quad \forall w_{d}\in U^{d}. $$

Then, by functional analysis (see [29]), there exists an extension $R^{h}: U\to U_{h}$ of $R^{d}$ satisfying $R^{h}|_{U_{h}}=R^{d}: U_{h}\to U^{d}$ and

38 $$ \bigl(\nabla R^{h}{ u}, \nabla{ w}_{h} \bigr)= (\nabla{ u}, \nabla{ w}_{h}),\quad\forall{ w}_{h}\in U_{h}, $$

where $u\in U$. Due to (38), the operator $R^{h}$ is bounded. We have

39 $$ \big\| \nabla\bigl(R^{h}{ u}\bigr)\big\| _{0}\leq\| \nabla{ u}\|_{0}, \quad\forall{u}\in U. $$

Further, the following holds.

#### Lemma 5

*For every* *d* ($1\leq d\leq l$), *the Ritz*-*operator* $R^{d}$ *in* (37) *satisfies*

40 $$ \frac{1}{ L }\sum_{i=1}^{L} \big\| \nabla\bigl({ u}_{h}^{i}-R^{d}{ u}_{h}^{i}\bigr)\big\| _{0}^{2}\leq\sum _{j=d+1}^{l}\lambda_{j}, $$

*where* $u_{h}^{i}\in\mathcal{V}$ ($i=1,2, \ldots, L$) *are the solutions to Problem* 4. *Further*, *if* $u\in H^{2}(\Theta)$ *is the solution to Problem* 2, *the extended Ritz*-*operator* $R^{h}$ *defined by* (38) *satisfies the following error estimations*:

41 $$\begin{aligned}& \big\| u -R^{h}u\big\| _{0}\leq Ch \big\| \nabla\bigl(u -R^{h}u\bigr)\big\| _{0},\quad\forall{ u}\in U, \end{aligned}$$

42 $$\begin{aligned}& \big\| u(t_{n}) -R^{h}u(t_{n}) \big\| _{s} \leq Ch^{k+1-s},\quad n=1, 2, \ldots, N, s=0,1. \end{aligned}$$

Thus, by means of $U^{d}$, the OFEE format for the 2D viscoelastic wave equation is described as follows:

#### Problem 6

Seek $u_{d}^{n}\in U^{d}$ ($n=1,2,\ldots,N$) satisfying

43 $$\begin{aligned}& u_{d}^{n} =R^{d}u_{h}^{n}= \sum_{j=1}^{d}\bigl(\nabla u_{h}^{n}, \nabla\psi_{j}\bigr) \psi_{j},\quad n=1,2,\ldots,L, \end{aligned}$$

44 $$\begin{aligned}& \begin{aligned}[b] & \frac{1}{\Delta t^{2}} \bigl(u_{d}^{n+1}-2u_{d}^{n}+u_{d}^{n-1},v_{d} \bigr)+\frac {1}{2\Delta t}\bigl(\nabla\bigl(u_{d}^{n+1}-u_{d}^{n-1} \bigr),\nabla v_{d}\bigr) +\frac{1}{2}\bigl(\nabla\bigl(u_{d}^{n+1}+u_{d}^{n-1}\bigr),\nabla v_{d}\bigr) \\ &\quad= \bigl(f^{n},v_{d}\bigr),\quad\forall v_{d}\in U^{d}, L\leq n\leq N-1, \end{aligned} \end{aligned}$$

where $u_{h}^{n}$ ($n=1, 2, \ldots, L$) are the first *L* solutions for Problem 4.

#### Remark 2

It is easily seen that Problem 4 at each time node includes $N_{h}$ unknowns (where $N_{h}$ is the number of vertices of triangles in $\Im_{h}$), whereas Problem 6 at the same time node contains only *d* unknowns ($d\ll l\leq L\ll N\ll N_{h}$). For real-world engineering problems, the number $N_{h}$ of vertices of triangles in $\Im _{h}$ can easily reach a few millions, while *d* is only the number of the major eigenvalues and is very small (for example, in Section 4, $d=6$, but $N_{h}\geq4\times10^{4}$). Problem 6 here is the OFEE format for the 2D viscoelastic wave equation. In particular, Problem 6 employs only the initial few known *L* solutions of Problem 4 used to extrapolate other $N-L$ solutions, and has no repetitive computations. The first *L* OFEE solutions are obtained by projecting the first *L* classical FE solutions into the POD basis, while the other remaining ($N-L$) OFEE solutions are obtained by extrapolating and iterating equation (44). Therefore, it is completely different from the existing POD-based reduced-order formulations.

### The error estimations of the OFEE solutions

In the following, we employ the classical FE method to deduce the error estimations of OFEE solutions for the 2D viscoelastic wave equation. We have the following main result.

#### Theorem 6

*Under the same conditions as Theorem* 3, *Problem* 6 *has a unique solution sequence* $\{u_{h}^{n}\}_{n=1}^{N}\subset U$ *satisfying*

45 $$ \big\| \nabla u_{d}^{n} \big\| _{0} \leq\Biggl(2{\beta^{-2}}\Delta t\sum_{i=1}^{N}\big\| f^{i}\big\| _{-1}^{2}+ 2\| \nabla\varphi_{0}\|_{0}^{2}+ \bigl(2+\beta^{-2}\bigr)\|\nabla\varphi_{1} \|_{1}^{2} \Biggr)^{1/2}. $$

*As a consequence*, *the sequence of solutions* $u_{d}^{n}$ *to Problem* 6 *is stable and continuously dependent on the source function* *f* *and the initial values* $\varphi_{0}$ *and* $\varphi_{1}$. *As* $h=O(\Delta t)$, *we have the following error estimations*:

46 $$ \big\| \nabla\bigl(u_{d}^{n}-u(t_{n}) \bigr)\big\| _{0}\leq C \Biggl[ \Biggl(L\sum_{j=d+1}^{l} \lambda_{j} \Biggr)^{1/2}+\Delta t^{2}+h^{k} \Biggr],\quad1\leq n\leq N. $$

#### Proof

(a) *The existence and uniqueness of solutions* $u_{d}^{n}$ *for Problem* *6*.

When $n=1,2, \ldots, L$, it is obvious that Problem 6 has a unique solution subset $\{u_{d}^{n}\}_{n=1}^{L}$ obtained by (43).

When $n=L+1,L+2, \ldots, N$, let

$$\begin{gathered} a\bigl(u_{d}^{n},v_{d}\bigr)= 2 \bigl(u_{d}^{n},v_{d}\bigr)+\Delta t\bigl(\nabla u_{d}^{n},\nabla v_{d}\bigr)+ \Delta t^{2}\bigl(\nabla u_{d}^{n}, \nabla v_{d}\bigr), \\F(v_{h})={\Delta t^{2}}\bigl(f^{n-1},v_{d} \bigr)+2\bigl(2u_{d}^{n-1}-u_{d}^{n-2},v_{d} \bigr)+{\Delta t}\bigl(\nabla u_{d}^{n-2},\nabla v_{d}\bigr)-{\Delta t}^{2}\bigl(\nabla u_{d}^{n-2}, \nabla v_{d}\bigr). \end{gathered} $$

Thus, (43) in Problem 6 can be rewritten as follows:

Seek $u_{d}^{n}\in U^{h}$ ($n=L+1,L+2,\ldots,N$) satisfying

47 $$ a\bigl(u_{d}^{n},v_{d} \bigr)=F(v_{d}),\quad\forall v_{d}\in U^{d}, n=L+1, L+2, \ldots, N. $$

It is obvious that, for given $u_{d}^{n-1}$ and $u_{d}^{n-2}$ as well as $f^{n-1}$ ($n=L+1,L+2, \ldots, N$), $F(v_{d})$ is a bounded linear functional of $v_{d}$ and $a(u,v)$ is a bilinear functional of *u* and *v*. Because $\|u\|_{0}\leq\|u\|_{1}$ and $\|\nabla u\|_{0}\leq\|u\|_{1}$, by using the Hölder inequality, we have

$$\begin{aligned}\big| a\bigl(u_{d}^{n},v_{d} \bigr)\big|&= \big|2\bigl(u_{d}^{n},v_{d}\bigr)+ \Delta t\bigl(\nabla u_{d}^{n+1},\nabla v_{d} \bigr)+ \Delta t^{2}\bigl(\nabla u_{d}^{n+1},\nabla v_{d}\bigr)\big| \\ &\leq2\big\| u_{d}^{n+1}\big\| _{0} \|v_{d}\|_{0}+\Delta t\big\| \nabla u_{d}^{n+1} \big\| _{0}\|\nabla v_{d}\|_{0}+ \Delta t^{2} \big\| \nabla u_{d}^{n+1}\big\| _{0}\|\nabla v_{d} \|_{0} \\ &\leq M\big\| u_{d}^{n+1}\big\| _{1} \|v_{d}\|_{1}, \end{aligned} $$

where $M=\max\{2, \Delta t, \Delta t^{2}\}$. Therefore, $a(u,v)$ is bounded on $U^{d}\times U^{d}$. Furthermore, we have

48 $$ \begin{aligned}[b] a(v,v)&= 2(v,v)+\Delta t( \nabla v,\nabla v)+ \Delta t^{2}(\nabla v,\nabla v) \\ &=2\|v\|_{0}^{2}+\Delta t\|\nabla v \|_{0}^{2}+ \Delta t^{2}\|\nabla v\| \\ &\geq\alpha\|v\|_{1}^{2},\quad\forall v\in U^{d}, \end{aligned} $$

where $\alpha=\min\{2, \Delta t, \Delta t^{2}\}$. Thus, $a(\cdot,\cdot )$ is positive definitive on $U^{h}\times U^{d}$. Therefore, by the Lax-Milgram theorem, for given $u_{d}^{n-1}$ and $u_{d}^{n-2}$, the system of equations (47) has a unique sequence of solutions $u_{d}^{n}$ ($n=L+1, L+2, \ldots, N$). Thus, Problem 6 has a unique sequence of solutions $u_{d}^{n}$ ($n=1,2, \ldots, L, L+1, \ldots, N$).

(b) *The stability of the sequence of solutions* $u_{d}^{n}$ *for Problem* *6*.

When $n=1, 2, \ldots, L$, by (43), (39), and (23) of Theorem 3, we obtain

49 $$ \begin{aligned}[b] \big\| \nabla u_{d}^{n} \big\| _{0}&=\big\| \nabla R^{d}u_{h}\big\| _{0}\leq\big\| \nabla u_{h}^{n}\big\| _{0} \\ &\leq\Biggl({\beta^{-2}}\Delta t\sum _{i=1}^{L}\big\| f^{i}\big\| _{-1}^{2}+ 2\|\nabla\varphi_{0}\|_{0}^{2}+\bigl(2+ \beta^{-2}\bigr)\|\nabla\varphi_{1}\|_{1}^{2} \Biggr)^{1/2}. \end{aligned} $$

For $n=L+1, L+2, \ldots, N$, by taking $v_{d}=u_{d}^{n}-u_{d}^{n-2}$ in (44) and using the Hölder, Poincaré, and Cauchy-Schwarz inequalities, we have

50 $$ \begin{aligned}[b] &\big\| u_{d}^{n}-u_{d}^{n-1} \big\| _{0}^{2}-\big\| u_{d}^{n-1}-u_{d}^{n-2} \big\| _{0}^{2}+\frac{\Delta t}{2}\big\| \nabla\bigl(u_{d}^{n}-u_{d}^{n-2} \bigr)\big\| _{0}^{2} +\frac{\Delta t^{2}}{2}\bigl(\big\| \nabla u_{d}^{n} \big\| _{0}^{2}-\big\| \nabla u_{d}^{n-2} \big\| _{0}^{2}\bigr) \\ &\quad\leq\frac{\Delta t^{3}}{2\beta^{2}}\big\| f^{n-1} \big\| _{-1}^{2}+\frac{\Delta t}{2}\big\| \nabla\bigl(u_{d}^{n}-u_{d}^{n-2} \bigr)\big\| _{0}^{2}. \end{aligned} $$

By summing (49) from $L+1$ to *n* and using the properties of the Ritz projection $R^{h}$ and (29), we obtain

51 $$ \begin{aligned}[b] &\big\| u_{d}^{n}-u_{d}^{n-1} \big\| _{0}^{2}+{\Delta t^{2}}\bigl(\big\| \nabla u_{d}^{n}\big\| _{0}^{2}+\big\| \nabla u_{d}^{n-1}\big\| _{0}^{2}\bigr) \\ &\quad\leq\frac{\Delta t^{3}}{\beta^{2}}\sum_{i=L+1}^{n} \big\| f^{i}\big\| _{-1}^{2}+2\Delta t^{2}\bigl(\big\| \nabla u_{d}^{L-1}\big\| _{0}^{2}+\big\| \nabla u_{d}^{L}\big\| _{0}^{2}\bigr) +\big\| u_{d}^{L}-u_{d}^{L-1}\big\| _{0}^{2} \\ &\quad\leq\frac{\Delta t^{3}}{\beta^{2}}\sum_{i=L+1}^{n} \big\| f^{i}\big\| _{-1}^{2} +2\Delta t^{2}\bigl(\|\nabla\varphi_{0}\|_{0}^{2}+\|\nabla\varphi_{1}\| _{0}^{2}\bigr)+\beta ^{-2}\Delta t^{2}\|\nabla\varphi_{1}\|_{0}^{2}. \end{aligned} $$

By combining (49) and (51), we immediately obtain (45).

(c) *The convergence of the sequence of solutions* $u_{d}^{n}$ *for Problem* *4*.

Let $\tilde{e}_{d}^{n}=u_{h}^{n}-u_{d}^{n}$, $E_{d}^{n}=R^{d}u_{h}^{n}-u_{d}^{n}$, and $\rho_{d}^{n}=u_{h}^{n}-R^{d}u_{h}^{n}$. By subtracting Problem 6 from Problem 4 and taking $v=v_{d}\in U^{d}$, we obtain the following system of error equations:

52 $$\begin{aligned}& \tilde{e}_{d}^{n}=u_{h}^{n}-u_{d}^{n}=u_{h}^{n}-R^{d}u_{h}^{n},\quad n=1,2, \ldots, L, \end{aligned}$$

53 $$\begin{aligned}& \begin{aligned}[b] &\frac{1}{\Delta t^{2}}\bigl(\tilde{e}_{d}^{n+1}-2 \tilde{e}_{d}^{n}+\tilde{e}_{d}^{n-1},v_{d} \bigr) +\frac{1}{2\Delta t}\bigl(\nabla\bigl(\tilde{e}_{d}^{n+1}-\tilde {e}_{d}^{n-1}\bigr),\nabla v_{d}\bigr) \\ &\quad{}+\frac{1}{2}\bigl(\nabla\bigl(\tilde{e}_{d}^{n+1}+ \tilde{e}_{d}^{n-1}\bigr),\nabla v_{d}\bigr) = 0,\quad \forall v_{d}\in U^{d}, n=L, L+1, \ldots, N-1. \end{aligned} \end{aligned}$$

For $n=1, 2, \ldots, L$, by (40) in Lemma 5 and (52), we have

54 $$ \big\| \nabla \tilde{e}_{d}^{n}\big\| =\big\| \nabla\bigl(u_{h}^{n}-u_{d}^{n} \bigr)\big\| _{0}=\big\| \nabla\bigl(u_{h}^{n}-R^{d}u_{h}^{n} \bigr)\big\| _{0} \leq\Biggl(L\sum_{j=d+1}^{l} \lambda_{j} \Biggr)^{1/2},\quad n=1,2, \ldots, L. $$

By combining (54) and (24), we obtain (46) for $n=1,2, \ldots, L$.

For $n=L+1, L+2, \ldots, N$, by the system of error equations (53) and the properties of the Ritz projection $R^{d}$, for $h=O(\Delta t)$, we have

55 $$ \begin{aligned}[b] &\big\| E_{d}^{n}-E_{d}^{n-1} \big\| _{0}^{2}-\big\| E_{d}^{n-1}-E_{d}^{n-2} \big\| _{0}^{2}+\frac{\Delta t}{2}\big\| \nabla\bigl(E_{d}^{n}-E_{d}^{n-2} \bigr)\big\| _{0}^{2} \\ &\qquad{}+\frac{\Delta t^{2}}{2}\bigl(\big\| \nabla E_{d}^{n} \big\| _{0}^{2}-\big\| \nabla E_{d}^{n-2} \big\| _{0}^{2}\bigr) \\ &\quad=-\bigl(\rho_{d}^{n}-2 \rho_{d}^{n-1}+\rho_{d}^{n-2},E_{d}^{n}-E_{d}^{n-2} \bigr) \\ &\quad\leq Ch^{-1}\bigl(\big\| \rho_{d}^{n} \big\| _{-1}^{2}+\big\| \rho_{d}^{n-1} \big\| _{-1}^{2}+\big\| \rho_{d}^{n-2} \big\| _{-1}^{2}\bigr) +\frac{\Delta t}{2}\big\| \nabla\bigl(E_{d}^{n+1}-E_{d}^{n-1}\bigr)\big\| _{0}^{2} \\ &\quad\leq Ch^{2k+3}+\frac{\Delta t}{2}\big\| \nabla \bigl(E_{d}^{n+1}-E_{d}^{n-1}\bigr) \big\| _{0}^{2}. \end{aligned} $$

By summing (32) from $L+1$ to *n*, and by (46) and (40) in Lemma 5, we obtain

56 $$ \begin{aligned}[b] &2\big\| E_{d}^{n}-E_{d}^{n-1} \big\| _{0}^{2}+{\Delta t^{2}}\bigl(\big\| \nabla E_{d}^{n}\big\| _{0}^{2}+\big\| \nabla E_{d}^{n-1}\big\| _{0}^{2}\bigr) \\ &\quad\leq C(n-L)h^{2k+3}+2\big\| E_{d}^{L}-E_{d}^{L-1} \big\| _{0}^{2}+{\Delta t^{2}}\bigl(\big\| \nabla E_{d}^{L}\big\| _{0}^{2}+\big\| \nabla E_{d}^{L-1}\big\| _{0}^{2}\bigr) \\ &\quad\leq C(\Delta t)^{2} \Biggl((n-L)h^{2k+1} +L\sum_{j=d+1}^{l}\lambda_{j} \Biggr). \end{aligned} $$

When $h=O(\Delta t)$, from (56) and by the properties of the Ritz projection and Theorem 3, we readily obtain the case of (46) when $n=L+1, L+2, \ldots, N$. □

#### Remark 3

We make some comments on Theorem 6:

1. It is known from Theorem 6 that, in order to not adversely affect accuracy, it is necessary to take *L* as $L\ll N$, for example, we usually take *L* such that $\sqrt{L}<5$. Thus, it is unnecessary to extract total transient solutions at all time nodal points $t_{n}$ as snapshots such as done in [19, 20].
2. The error $(L\sum_{j=d+1}^{l}\lambda_{j} )^{1/2}$ in Theorem 6 gives some indication as to how to choose the number *d* of the POD basis, namely, it is only necessary to meet
  $$\Biggl(L\sum_{j=d+1}^{l} \lambda_{j} \Biggr)^{1/2}\leq\max\bigl\{ \Delta t^{2}, h^{k}\bigr\} . $$

### The implement procedure of the OFEE format

Solving the OFEE format, *i.e.*, Problem 6, requires the following seven steps:

*Step 1*. For given *ε* and *γ*, boundary value function $\varphi(x,y,t)$, initial value function $\varphi_{0}(x,y)$, and $\varphi_{1}(x,y)$, source term $f(x,y,t)$, the time step size Δ*t*, and the spatial grid measurement *h* satisfying $h=O(\Delta t)$ solve the following classical FM formulation on the first *L* ($\sqrt {L}<5$) steps:

$$ \begin{gathered} \frac{1}{\Delta t^{2}}\bigl(u_{h}^{n+1}-2u_{h}^{n}+u_{h}^{n-1},v_{h} \bigr)+\frac {1}{2\Delta t}\bigl(\nabla\bigl(u_{h}^{n+1}-u_{h}^{n-1} \bigr),\nabla v_{h}\bigr) \\ \quad{}+\frac{1}{2}\bigl(\nabla\bigl(u_{h}^{n+1}+u_{h}^{n-1} \bigr),\nabla v_{h}\bigr) = \bigl(f^{n},v_{h} \bigr), \quad\forall v_{h}\in U_{h}, 1\leq n\leq N-1, \\ u_{h}^{0}=R_{h} \varphi_{0}(x, y),\quad\quad u_{h}^{1}=R_{h}\bigl( \varphi_{0}(x, y)\bigr)+\Delta tR_{h}\bigl( \varphi_{1}(x,y)\bigr), \quad(x, y)\in\Theta, \end{gathered} $$

where $f^{n}=f(t_{n})$ and $R_{h}$ is the Ritz projection. This yields the snapshots $W_{i}=u_{h}^{n}$ ($n=1,2,\ldots, L$).

*Step 2*. Formulate the snapshot matrix $\boldsymbol {A}=({A}_{ij})_{L\times L}$, where ${A}_{ij}=(\nabla u_{h}^{i}, \nabla u_{h}^{j})$ and $(\cdot,\cdot)$ is the $L^{2}$-inner product.

*Step 3*. Find the eigenvalues $\lambda_{1}\geq\lambda_{2}\geq \cdots\geq\lambda_{l}>0$ ($l=\operatorname{dim}\{u_{h}^{n}: 1\leq n\leq L\}$) of ***A*** and the corresponding eigenvectors $\boldsymbol {v}^{j}=(a_{1}^{j},a_{2}^{j},\ldots, a_{L}^{j})$ ($j=1,2,\ldots,l$).

*Step 4*. For the error $\delta=O(\Delta t^{2},h^{k})$ needed, decide the number *d* of the POD basis satisfying $(L\sum _{j=d+1}^{l}\lambda_{j})^{1/2}\leq\delta$.

*Step 5*. Produce the POD basis $\psi_{j}=\sum _{i=1}^{L}a_{i}^{j}u_{h}^{i}/{\sqrt{L\lambda_{j}}}$ ($j=1,2,\ldots,d$).

*Step 6*. Solve the following system of equations with *d* degrees of freedom at each time node:

$$ \begin{gathered} u_{d}^{n} =R^{d}u_{h}^{n}=\sum _{j=1}^{d}\bigl(\nabla u_{h}^{n}, \nabla\psi_{j}\bigr)\psi_{j},\quad n=1,2,\ldots,L, \\ \frac{1}{\Delta t^{2}}\bigl(u_{d}^{n+1}-2u_{d}^{n}+u_{d}^{n-1},v_{d} \bigr)+\frac {1}{2\Delta t}\bigl(\nabla\bigl(u_{d}^{n+1}-u_{d}^{n-1} \bigr),\nabla v_{d}\bigr) \\ \quad{}+\frac{1}{2}\bigl(\nabla\bigl(u_{d}^{n+1}+u_{d}^{n-1} \bigr),\nabla v_{d}\bigr) = \bigl(f^{n},v_{d} \bigr), \quad\forall v_{d}\in U^{d}, L\leq n\leq N-1, \end{gathered} $$

to attain the OFEE solutions $u_{d}^{n}$ ($n=1,2,\ldots,N$).

*Step 7*. If $\|u_{d}^{n-1}- u_{d}^{n}\|_{1}\geq\| u_{d}^{n}-u_{d}^{n+1}\|_{1}$ ($n=L, L+1,\ldots, N-1$), then $u_{d}^{n}$ ($n=1,2, \ldots, N$) are the OFEE solutions for Problem 6 satisfying the desired accuracy. Else, *i.e.*, if $\|u_{d}^{n-1}- u_{d}^{n}\|_{1} <\|u_{d}^{n}- u_{d}^{n+1}\|_{1}$ ($n=L, L+1,\ldots, N-1$), let $W_{i}=u_{d}^{i}$ ($i=n-L, n-L+1, \ldots, n-1$) and return to *Step 2*.

#### Remark 4

Though the OFEE solutions of Problem 6 are theoretically ensured with an accuracy of order $O(\Delta t^{2},h^{k})$ (if $\Delta t=O(h)$), due to error accumulation in the computational process, the actual numerical solutions may contain a larger error than theoretically predicted. Therefore, in order to obtain numerical solutions with the desired computing accuracy, it is best to add Step 7; if the computing accuracy is unsatisfactory, improvements of numerical solutions can be made by renewing the snapshots and the POD basis. This explains why the OFEE format is superior to the classical SPDMFE method.

## Numerical simulations

In this section, we furnish a numerical example to illustrate that the results of numerical computation are concordant with our theoretical analysis and also demonstrate the feasibility and efficiency of the OFEE format for the 2D viscoelastic wave equation.

The computational domain is irregular and consists of a set $\overline {\Theta}=([0, 2]\times[0,2])\cup([0.65, 1.3]\times[2, 2.03])\mbox{ cm}^{2}$. The source term is taken as $f(x,y,t)=0$ and the initial and boundary value functions are taken as follows, for $0\leq t\leq T$:

$$\varphi(x,y,t)= \varphi_{0}(x,y)= \varphi_{1}(x,y)= \left\{ \textstyle\begin{array}{l@{\quad}l} 2-x, &\hbox{if } (x,y)\in[1.5, 2]\times[2, 2],\\ 0.5, &\hbox{if } (x,y)\in[0.65, 1.5]\times[2, 2.03],\\ 0.0, &\hbox{others}. \end{array}\displaystyle \right . $$

Thus, $\varphi_{0}(x,y)$ and $\varphi_{1}(x,y)$ all are almost everywhere differentiable on Θ̄ and their first-order partial derivatives are almost everywhere zero on Θ̄.

We first divide the domain Θ̄ into $200\times200$ small squares with side length $\triangle x=\triangle y=10^{-2}$. Then we link the diagonal of the square to divide each square into two triangles and each in the same direction. Further, we adopt local refining meshes such that the scale of meshes on $[0.65, 1.3]\times[2, 2.03]$ and nearby $(x,2)$ ($0\leq x \leq2$) are one-third of the meshes nearby $(x,0)$ ($0\leq x \leq2$), forming the triangularization $\Im_{h}$. Thus $h=\sqrt{2}\times 10^{-2}$. In order to satisfy $k=O(h)$, we take the time step size $k=10^{-2}$. The MFE space $U_{h}$ is taken as piecewise linear polynomials.

**Figure 1:**
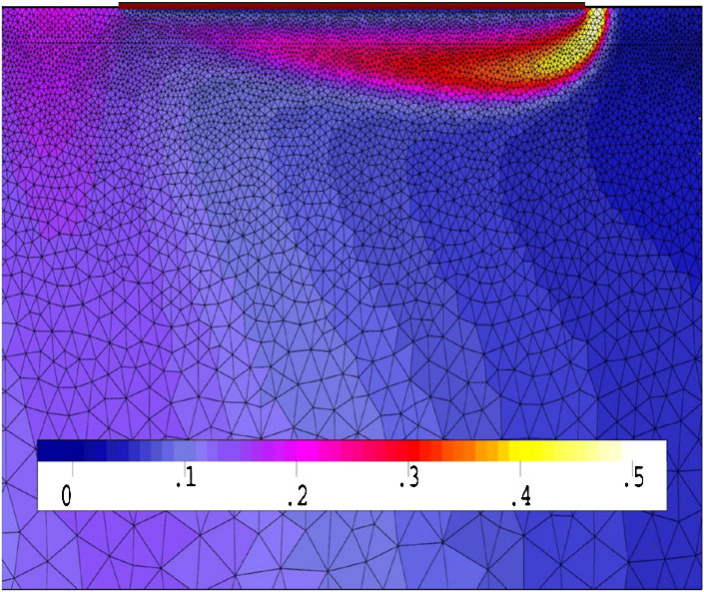
**Contour plot of classical FE solution** $\pmb{u_{h}^{n}}$ **at** $\pmb{t=2}$ **.**

**Figure 2:**
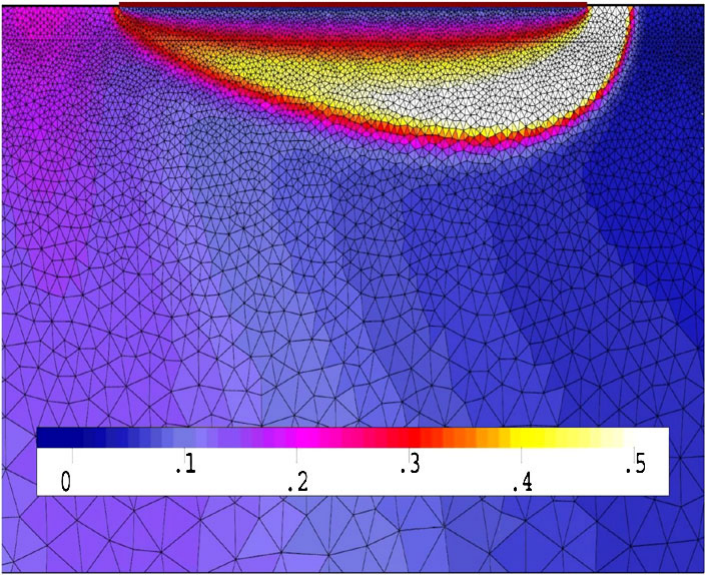
**Contour plot of OFEE solution** $\pmb{u_{d}^{n}}$ **at** $\pmb{{t=2}}$ **.**

**Figure 3:**
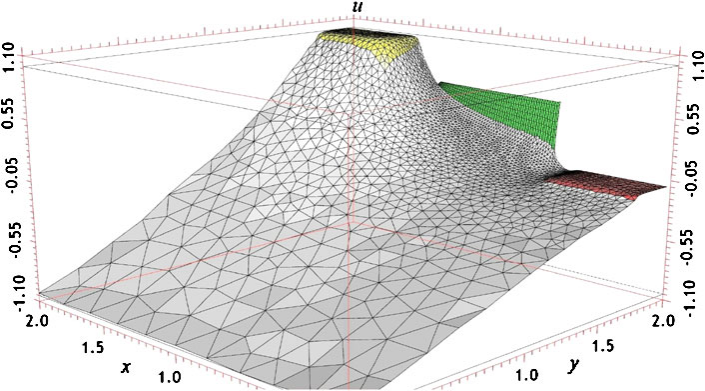
**3D graphical image of classical FE solution** $\pmb{u_{h}^{n}}$ **at** $\pmb{t=2}$ **.**

**Figure 4:**
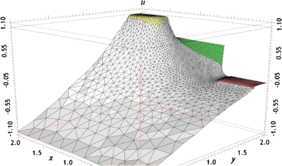
**3D graphical image of OFEE solution** $\pmb{u_{d}^{n}}$ **at** $\pmb{t=2}$ **.**

We have found the numerical solutions $u_{h}^{n}$ with the classical FE formulation (Problem 4) when $t=2$, depicted graphically in Figures 1 and 3. We choose the first 20 solutions $u_{h}^{n}$ ($n=1,2,\ldots, 20$, *i.e.*, at time $t=0.01, 0.02, \ldots, 0.2$) for Problem 4 (the classical FE formulation) to constitute a set of snapshots. By computing, with $d=6$ and $k=10^{-2}$, we achieve the error estimation $(20\sum_{j=7}^{20}\lambda_{j})^{1/2}\leq4\times10^{-4}$ in Theorem 6, which shows that we only need to take six POD bases. Thus, the OFEE format (Problem 6) at each time level has only 6 degrees of freedom, while the classical FE formulation (Problem 4) contains more than $4\times10^{4}$ degrees of freedom. Therefore, the OFEE format (Problem 6) cannot only alleviate the computational load and save time-consuming calculations in the computational process, but also reduce the accumulation of truncation errors in the computational process. When we solve the OFEE format (Problem 6) with six optimal POD bases, according to the seven steps of implementation of the OFEE format in Section 3.3, we find that the OFEE format at $t=2$ is still convergent, without the need to renew the POD basis. The OFEE solution obtained with the OFEE format (Problem 6) is depicted graphically in Figures 2 and 4. The images in Figures 1 and 2 look very much alike, and so do those in Figures 3 and 4. Nevertheless, the OFEE solutions are probably better than the classical FE solutions due to the little accumulation of truncated errors of the OFEE format (Problem 6) in the computational process.

**Figure 5:**
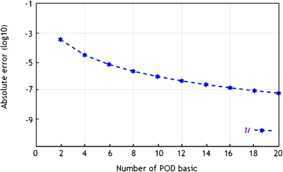
**When** $\pmb{t=2}$ **, the absolute errors between the solutions of Problem** **6** **with different numbers of POD bases for a group of 20 snapshots and the classical FE formulation Problem** **4** **with piecewise first-degree polynomial.**

Figure 5 shows the absolute error between 20 solutions $u_{d}^{n}$ of the OFEE format (Problem 6) with 20 different numbers of POD bases and the solutions $u_{h}^{n}$ of the classical FE formulation (Problem 4) at $t=2$. It shows that, when the numbers of the POD basis are larger than five, the error does not exceed $4\times10^{-4}$. Therefore, the error results in the numerical example above are concordant with those obtained with the theoretical approach. This has shown that the OFEE format is feasible and efficient for solving the viscoelastic wave equation.

## Conclusions

In this article, we use the POD technique to build the OFEE format for the 2D viscoelastic wave equation. We first extract snapshots from the initial few *L* ($L\ll N$) classical FE solutions for the 2D viscoelastic wave equation. Next, we constitute the POD basis of snapshots by means of the POD method. Then the FE subspaces of the classical FE format are replaced with the subspaces spanning the most main POD bases to build the OFEE formulation for the 2D time-dependent conduction-convection problem. Finally, we deduce the existence, uniqueness, stability, and convergence of the OFEE solutions of the 2D viscoelastic wave equation and furnish the implement procedure for the OFEE format. Comparing the numerical simulation errors with the theoretical errors we have verified that the theoretical errors are concordant with the computing errors, thus validating both the feasibility and efficiency of the OFEE format.
